# Maturation of infant sleep during the first 6 months of life: a mini-scoping review

**DOI:** 10.3389/fnins.2025.1581325

**Published:** 2025-04-30

**Authors:** Abriana Gilchrist, Brandon S. Aylward, Christopher M. Laine, Harvey Karp

**Affiliations:** ^1^T.H. Chan Division of Occupational Science and Occupational Therapy, University of Southern California, Los Angeles, CA, United States; ^2^Happiest Baby Inc., Los Angeles, CA, United States

**Keywords:** sleep, infant sleep, total night sleep, total night awakenings, longest sleep stretch, actigraphy, sleep diary, sleep monitoring

## Abstract

**Introduction:**

Several foundational aspects of neurodevelopment occur during the early months of infant life, most notably the maturation and consolidation of wake/sleep cycles. Past studies have had difficulty quantifying infant sleep, with most researchers relying on low-resolution caregiver surveys. Data obtained from nightly measurements have not yet been aggregated across studies to clarify developmental trajectories and population norms. This mini-scoping review assesses data collected from actigraphy and sleep diaries; the two most common nightly infant sleep measurement techniques.

**Methods:**

The PubMed database was used to identify studies from 2000 to 2024 utilizing actigraphy and/or sleep diaries, and which report total night sleep (TNS), longest sleep stretch (LSS), and/or frequency of night wakings (NW) during the first 6 months of life. Data was then compiled per metric to reveal the extent of inter-and intra-study variability, and curves were fit to highlight developmental trajectories.

**Results:**

A total of 35 articles met inclusion criteria (16 studies using actigraphy only, 8 studies using sleep diary only, and 11 studies using both actigraphy and sleep diaries). The sample sizes of these studies ranged from 13 to 320 infants. The majority of studies (*N* = 28) reported two or fewer age data points.

**Conclusion:**

Aggregation and regression revealed longitudinal trends, but highlighted variability within and between studies, as well as systematic differences between measurement methods. In order to establish reliable benchmarks, future studies must include well defined, objective measures of sleep as well as greater methodological consistency, larger cohorts, more frequent sampling, and clear disclosure of methodological limitations.

## 1 Introduction

Sleep during infancy is important for the well-being of both the infant and caregiver. The neurophysiology and maturation of sleep are linked to neuronal organization (including language development, memory, and architectural maturation) in infants and children (Schoch et al., 2021; Colombo et al., 2021; Lokhandwala and Spencer, 2022; Donnici et al., 2023). Disrupted or reduced infant sleep has also been linked to increased maternal stress and depression (Owais et al., 2018; Sharkey et al., 2016; Dennis and Ross, 2005).

Infant sleep–wake cycles are diverse across infants and they undergo nocturnal consolidation and maturation over the first 6 months of age (Colombo et al., 2021; Adams et al., 2019). Over the first 6 months of life, infants demonstrate a progressive increase in total nocturnal sleep and longest continuous nocturnal sleep period and a progressive decrease in night wakings (Ednick et al., 2009; Mindell et al., 2016). Over these early months, sleep consolidation is often punctuated by setbacks related to growth spurts, illness, teething, and achievement of new developmental milestones. Consistent diurnal sleep and wake patterns typically develop between 3 to 6 months of age (Colombo et al., 2021), however 20–30% of children demonstrate sleep–wake problems that can persist through infancy and even into toddlerhood, negatively impacting infant and caregiver wellbeing (Sharkey et al., 2016; Tikotzky et al., 2023; Mindell and Lee, 2015; Mindell et al., 2017).

Measuring infant sleep is an important indicator of neurodevelopment (Tikotzky et al., 2023; Schoch et al., 2020), but inconsistencies in the definitions and data collection methodologies used undermine the generalizability of past reports. For example, hours of sleep per night will vary depending on how ‘night’ is defined, i.e., is it measured with respect to infant behavior (Pisch et al., 2019), caregiver behavior (Stremler et al., 2013), or fixed/imposed schedules (Shinohara and Kodama, 2012). The methodology used can also impact the reliability of the data collected (Tikotzky et al., 2023; Camerota et al., 2018) as well as the validity of comparisons made across methods (Adams et al., 2019) and studies (Alamian et al., 2016). Even within a single study, some have advocated for definitions based on composite measurements (Staples et al., 2019), since sleep is characterized not only by quantity, but also quality, timing of onset, duration, and consolidation, which evolve at different rates across infancy (Schoch et al., 2020; Staples et al., 2019).

Polysomnography (PSG) is generally considered the “gold standard” in sleep research, as it provides direct physiological monitoring. However it is expensive, and challenging to use with infants (Tikotzky et al., 2023). Automated videosomnography (Tikotzky et al., 2023; Kahn et al., 2021a; Kahn and Gradisar, 2022; Kahn et al., 2023; Kahn et al., 2021b) has captured recent interest as an objective assessment of infant sleep, however, large scale validations against other methods are lacking. For those reasons, most quantitative assessments of infant sleep at home have relied on two methodologies: parent diaries and actigraphy. Parent diaries are inexpensive and easy to administer, however they are completely subjective and prone to error (such as memory lapses and the underreporting of night waking in the absence of audible fussing). Actigraphy can have high sensitivity and is both cost-efficient and objective. However, defining sleep according to body movement is also prone to error (such as contamination from external motion, inaccuracy of movement thresholds in detecting wakefulness, equating stillness with sleep, etc.) and it is lacking standardization of interpretation (Schoch et al., 2021; Colombo et al., 2021; Schoch et al., 2019).

Consequently, confidence in sleep data collected using these two measures must be tempered by an understanding of methodological variations and by practical limitations on sample sizes and longitudinal data collection. To achieve a more accurate sleep assessment, some have argued that the combination of actigraphy and diaries may be preferable to relying on either method alone (Meltzer et al., 2012). Furthermore, aggregation of data across studies derived from these two methods may clarify the extent of variability within and between studies and reveal more accurate developmental sleep trajectories.

To investigate the potential value of analyzing an aggregation of quantitative infant sleep studies, we conducted a mini-scoping review of studies using either diaries or actigraphy or both measures to evaluate sleep over the first 6 months of life. Specifically, we aimed to characterize and assess: (1) the developmental trajectories of the most common sleep metrics: longest sleep stretch (LSS), total night sleep (TNS), and frequency of night wakings (NW), and (2) the nature and extent of variability that could impact comparison of these two methodologies across studies.

## 2 Methods

A mini-scoping review was completed to identify research papers from 01/01/2000–06/20/2024 that utilized actigraphy or daily journaling to measure longest sleep stretch, night wakings, and total duration of nightly sleep among infants.

### 2.1 Search methods

The initial search was conducted using the PubMed database, with the following advanced search query:

[Subject, Topic, and Method…]

("Infant"[Mesh] OR "infant sleep"[tiab])

AND

("Sleep"[Mesh] OR "Sleep Quality"[Mesh] OR "Sleep Hygiene"[Mesh] OR "infant sleep"[tiab])

AND

("Actigraphy"[Mesh] OR "Surveys and Questionnaires"[Mesh] OR "Sleep Latency"[Mesh] OR "Sleep Duration"[Mesh] OR "Polysomnography"[Mesh] OR "videosomnography"[tiab] OR "wakings"[tiab] OR "awakenings"[tiab] OR "longest sleep"[tiab] OR "sleep duration"[tiab])

[Other Filters…]

AND (excludepreprints[Filter])

AND (classicalarticle[Filter] OR clinicalstudy[Filter] OR clinicaltrial[Filter] OR controlledclinicaltrial[Filter] OR multicenterstudy[Filter] OR observationalstudy[Filter] OR pragmaticclinicaltrial[Filter] OR randomizedcontrolledtrial[Filter] OR researchsupportnihextramural[Filter] OR researchsupportnihintramural[Filter] OR researchsupportnonusgovt[Filter] OR researchsupportusgovtnonphs[Filter] OR researchsupportusgovtphs[Filter] OR researchsupportusgovernment[Filter])

AND (fft[Filter])

AND (humans[Filter])

AND (2000/1/1:2024/6/20[pdat])

AND (english[Filter])

AND (allchild[Filter] OR newborn[Filter] OR allinfant[Filter] OR infant[Filter])

NOT ("Review"[Publication Type] OR "systematic review"[Publication Type] OR "Meta-Analysis"[Publication Type])

Two authors reviewed each study and extracted relevant data. A third author reviewed data to identify and eliminate any discrepancies. Data extracted included bibliographic data, study sample size, data collection week, and objective measurement method(s) utilized. To identify additional sources of data, the references within each included study were compiled and searched for possible inclusion as well.

### 2.2 Curve fitting

A 2nd order robust regression was completed using an iteratively reweighted least squares (IRLS) approach, with reweighting by absolute residuals (Schlossmacher, 1973). The method uses a standard least squares fit procedure for an initial fit, then conducts a weighted least squares regression using 1/max[0.001, abs(residuals)] as the weights. The 0.001 is a regularization to prevent division by zero and set a maximal weight. This process iterates until error stabilizes, in this case when the RMS-error changes less than 0.001%. Other methods, such as direct outlier removal or weighting each study on the basis of sample size, study duration, cross-subject variability, or perceived quality would be problematic for the present dataset because (1) within-study error was not consistently quantified, (2) it is unlikely that the expected error would be the same across ages, (3) sample size and study duration would not clearly relate to the measurement precision or accuracy, and (4) altered definitions of night boundaries and analysis thresholds could impose systematic differences between studies which cannot meaningfully be normalized prior to aggregation. The curve fitting method utilized here avoids these issues by tracking the bulk of available data, assuming only relatively smooth change in metrics over time. Higher order fits could produce moderate improvement, but risk imposing artificial structure to the data and generally overfitting. It is also important to note that the constructed curves are not intended to have predictive power for individual infants, but rather to represent hypothetically ‘typical’ values at any given point in time. To quantify the overall spread of data around the curve, the 25th and 75th percentile of residuals were calculated.

Several studies reported data for multiple subpopulations. Pennestri et al. (2020) and Vijakkhana et al. (2015) reported TNS from sleep diaries grouped into those meeting a 6 hour criterion or 8 hour criterion. Vijakkhana et al. (2015) reported weekday versus weekend sleep. Rudzik et al. (2018) reported sleep for infants who were breastfed vs. formula fed, denoted via maternal reports. Such subgroups have been included here as distinct data points in scatterplots, given that within-study aggregation prior to curve fitting would not necessarily improve overall accuracy. Additionally, Colombo et al. (2021), Shinohara and Kodama (2012), Rudzik et al. (2018) collected data from babies with ages specified with longer than 1-week resolution. Data from these studies was spread over the specified ages (smaller circles in the scatter plots), as this allows the curve fitting method to spread the influence of the study more evenly over the age range in question. Similarly, the curve fitting approach reduces over-emphasizing studies with subgroups since weights are reset after considering all trajectory residuals together.

## 3 Results

A total of 800 studies were initially identified based on inclusion/exclusion criteria. The full texts of these articles were assessed for eligibility resulting in the identification of 25 articles. These 25 articles cited a total of 1,108 articles and of those, a total of 10 additional articles met inclusion criteria. Of the total 35 articles included in the scoping review, 16 studies used actigraphy only, 8 used sleep diaries only, and 11 used both actigraphy and sleep diaries to assess sleep. Across articles using actigraphy exclusively, 100% reported on TNS (*N* = 16), 56.3% on LSS (*N* = 9), and 50.0% on NW (*N* = 8). Across articles using sleep diaries exclusively, 75.0% reported on TNS (*N* = 6), 50.0% on LSS (*N* = 4), and 75.0% on NW (*N* = 6). Of the studies using both actigraphy and sleep diaries, 81.8% reported on NW (*N* = 9), 90.9% reported TNS (*N* = 10), and 45.5% collected data on LSS (*N* = 5). The average number of metrics reported in each study ranged from 1 to 6 (*M* = 2.64). Finally, the total number of timepoints reported in these studies ranged from 1 to 8 across the 24 weeks (*M* = 1.94). All included studies (Colombo et al., 2021; Adams et al., 2019; Pisch et al., 2019; Stremler et al., 2013; Shinohara and Kodama, 2012; Camerota et al., 2018; Pennestri et al., 2020; Vijakkhana et al., 2015; Rudzik et al., 2018; Ball, 2003; Cubero et al., 2005; Figueiredo et al., 2017; Galland et al., 2016; Galland et al., 2017; Guyer et al., 2015; Hauck et al., 2018; Konrad et al., 2016; Quillin and Glenn, 2004; Santos et al., 2019; Scher et al., 2004; Scher and Cohen, 2015; Spruyt et al., 2008; Sweeney et al., 2020; St James-Roberts and Gillham (2001); Stremler et al., 2006; Symon et al., 2005; Tikotzky and Sadeh, 2009; Tikotzky et al., 2010; Tikotzky et al., 2015; Tsai et al., 2018; Tsai et al., 2022; Volkovich et al., 2015; Volkovich et al., 2018; Yu et al., 2021) are listed in Table 1, along with a description of which measurements were made and at which timepoints. Further information on the search flow, descriptions of included articles, and the numerical data extracted from each study can be found in Supplementary material.

**Table 1:** Information about included studies.

Authors	Sample size	Timepoint(s) (wks)	Act TNS	Act LSS	Act NW	Diary TNS	Diary LSS	Diary NW
Adams et al. (2019)	24	6, 15, 24	X					
Ball (2003)	253 at 4wks; 248 at 12wks	4, 12						X
Camerota et al. (2018)	82 act.; 87 diary	12	X	X	X			X
Colombo et al. (2021)	66	2–5, 10–11, 16–17	X	X	X			
Cubero et al. (2005)	16	12	X	X				
Figueiredo et al. (2016)	94	2, 12, 24				X	X	X
Figueiredo et al. (2017)	148 at 2wks; 162 at 12wks; 123 at 24wks	2, 12, 24				X	X	X
Galland et al. (2016)	39	24	X		X	X		X
Galland et al. (2017)	209	24			X	X	X	X
Guyer et al. (2015)	14 term; 34 preterm	5, 11				X	X	
Hauck et al. (2018)	22	24	X					
Konrad et al. (2016)	24	24	X		X			
Pennestri et al. (2020)	44	24					X	X
Pisch et al. (2019)	40	16, 24	X		X			
Quillin and Glenn (2004)	33	4				X		X
Rudzik et al. (2018)	29	4, 6, 8, 10, 12, 14, 16, 18	X	X	X	X	X	X
Santos et al. (2019)	230	12, 24	X		X	X		X
Scher et al. (2004)	50 at 12 wks; 37 at 24wks	12, 24	X	X	X			
Scher and Cohen (2015)	26	20, 24	X		X			
Shinohara and Kodama (2012)	26	4–6,8–10, 14–16	X					
Spruyt et al. (2008)	17 at 12wks; 18 at 24wks	12, 24	X			X		
St James-Roberts and Gillham (2001)	203	3, 6, 12				X		X
Stremler et al. (2006)	15	6	X	X	X			
Stremler et al. (2013)	103 at 6wks; 102 at 12wks	6, 12	X	X	X			
Sweeney et al. (2020)	20	6, 12	X	X				
Symon et al. (2005)	121 at 6wks; 107 at 12wks	6, 12	X	X				
Tikotzky and Sadeh (2009)	85	24	X		X			X
Tikotzky et al. (2010)	96	24	X		X			
Tikotzky et al. (2015)	56 at 12wks; 54 at 24wks	12, 24	X		X			X
Tsai et al. (2018)	219	24	X			X	X	X
Tsai et al. (2022)	320	24	X					
Vijakkhana et al. (2015)	208	24				X		
Volkovich et al. (2015)	127 at 12wks; 114 at 24wks	12, 24	X	X	X			X
Volkovich et al. (2018)	127 at 12wks; 121 at 24wks	12, 24	X	X	X			X
Yu et al. (2021)	306	4, 24	X	X	X			

Sample sizes refer only to any non-intervention groups utilized in this review. TNS = total night sleep, LSS = longest sleep stretch, NW = night wakings. Act = actigraphy, Diary = nightly sleep journal.

**Figure 1:**
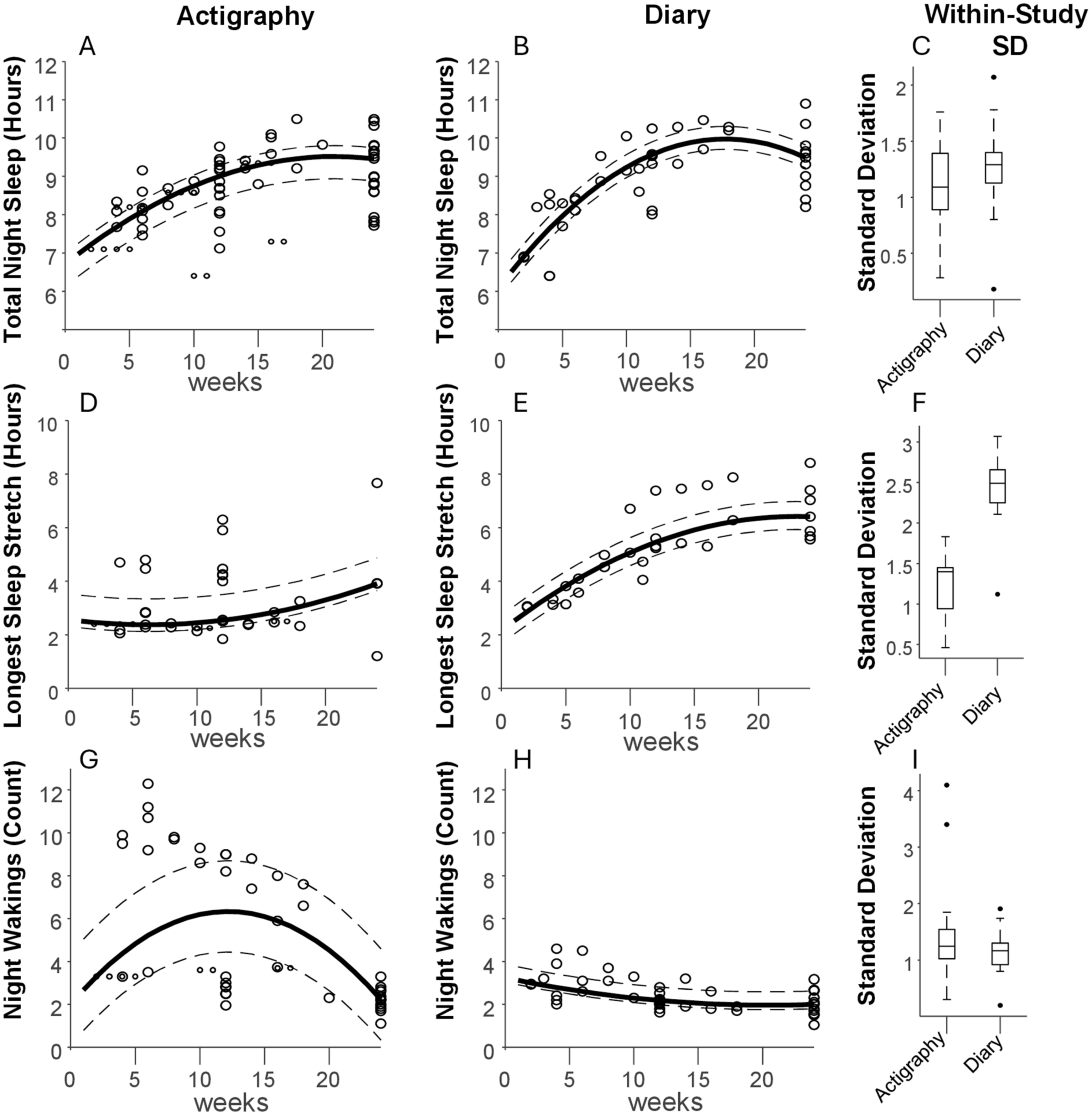
Aggregate sleep metrics. (From Left to Right) Panels **(A,B)** depict the total sleep at night measured in 27 studies using actigraphy and 11 using sleep diaries, respectively, during the first 6 months of life. Each data point is a measurement associated with a given week of life. A curve (solid line) was fit to the data to display the general trend, with dotted lines representing the 25th and 75th percentiles of the fit residuals (see Methods). The box-and-whisker plots in panel **(C)** show the spread of reported standard deviations per measurement. Panels **(D–F)** show the same analyses but for longest sleep stretch, and panels **(G–I)** show the same for number of nightly wakings.

The data extracted for each method and timepoint is summarized visually in Figure 1 for TNS, LSS, and NW. For TNS, the curve fit residuals had IQRs boundaries of −34.8 to 17.0 min for actigraphy and −16.1 to 19.9 min for diary. For LSS IQRs spanned from −15.1 to 57.8 min for actigraphy, and −29.4 to 33.3 min for diary. For NW, the IQRs ranged from −1.9 to 2.4 for actigraphy and from −0.2 to 0.6 for diary. In this case, the fitting procedure produced a curve that fell in between the different clusters of actigraphy data, producing a poor overall fit compared to data from sleep diaries. These residuals serve as a rough metric of between-study variability that can be expected at any given point in time. The dotted lines surrounding each curve represent the IQR of residuals around the fit curve. Within-study standard deviations are summarized by the box and whisker plots associated with each measure (Panels C, F, and I). Most studies reported standard deviation (see Supplementary material for details).

## 4 Discussion

To our knowledge, this is the first analysis of sleep metrics aggregating data from the most common nightly-monitoring approaches (actigraphy and sleep diaries) over the first 6 months of life. This review characterizes developmental trajectories of infant sleep, while also highlighting the variability within and between studies published over the past ~20 years. Interpretation of these studies must be undertaken with careful attention to differences in methods and definitions.

### 4.1 Total night sleep

In line with previous reviews (Galland et al., 2012), TNS is one of the more commonly reported measurements of sleep. In the current analysis, it was reported in 33 of 35 articles. For TNS, actigraphy and sleep diary results showed similar trajectories, starting at about 7–8 h per night in the first 4 weeks, increasing steadily until about 15–20 weeks, and converging to about 9.5 h at week 24.

Within-study variability, pooled across ages, was similar for actigraphy and diary, although somewhat higher for diary (median of = 1.1 vs. 1.3 h, respectively). Several studies reported an average TNS duration of over 10 h at 24 weeks of age, but these had relatively small sample sizes of 40 infants or less (Pisch et al., 2019; Konrad et al., 2016). Sleep diary studies generally reported higher TNS after the first month compared with actigraphy studies. For example, at week 16, diary studies showed ~10 h of TNS vs. 9.4 h in actigraphy studies. Though both curves converged by week 24, TNS at that time varied highly across studies, ranging from about 8 to 11 h.

### 4.2 Longest sleep stretch

This metric varied very widely between actigraphy and diary studies. The curve based on actigraphy showed a steady LSS of ~2.5 h until 10 weeks of age, increasing thereafter to about 4 hours at 24 weeks. The curve based on diaries also begins at 2.5 h, but then steadily increasing to ~6.5 h by week 24. Within-study variability was much higher for actigraphy vs. diary studies (median of 1.4 vs. 2.5 h, respectively). This may relate to how wakings are monitored. For diaries, feeding or observed fussiness would typically be required to note an awakening. For actigraphy, movement may exceed the waking threshold prior such obvious behaviors. However, active sleep can also involve substantial movement (Knoop et al., 2021). Thus, shorter LSS durations may represent detection of non-alerting awakenings and/or misattribution to wakefulness of motion occurring during ‘active sleep.’ Longer LSS values for actigraphy (well above the curve) may represent threshold alignment with noticeable behaviors, as they were often within range of what was reported using diaries at the same ages. The imprecise association of movement to waking is a known confounder for infant actigraphy, and reporting from actigraphy studies is often poor with respect to epoch length, algorithm, artifact identification, data loss and operational definition of sleep variables (Schoch et al., 2021). In fact, two actigraphy algorithms commonly used in pediatric sleep research (Sadeh and Oakley algorithms) (Meltzer et al., 2012; Sadeh et al., 1995; Weiss et al., 2010) have only moderate agreement (Schoch et al., 2019). In more than half of actigraphy validation studies, wakefulness specificity is under 60% (Meltzer et al., 2012).

### 4.3 Night wakings

NW showed the greatest discrepancy when comparing actigraphy vs. diaries. For actigraphy, the fitted trajectory for the frequency of NW resembled an inverted u-shape with an increase in wakings until approximately 12 weeks and a decrease thereafter. The shape itself emerges from the use of a second order fit to describe data that fall into distinct groups. One group of actigraphy studies reported ~2–3 wakings per night over all 6 months, similar to diaries, while another group describes a decreasing trend from about 10 wakings per night at week 5 to ~5 over the next few months. For diaries, the number of NW slowly decreased from about 3 to 2 per night between birth and 16 weeks, with little change between 16 and 24 weeks.

As with LSS, the larger numbers reported in the actigraphy studies may reflect non-alerting awakenings and/or misattribution of movement during ‘active sleep’ to awakenings. Studies reporting smaller numbers may have had closer alignment between threshold crossings and alerting behaviors. Similar issues appeared in Rudzik et al. (2018) who reported that NWs ranged from 5.9 to 10.7 reported using actigraphy, yet only 1.7 to 4.6 with sleep diaries. Stremler et al. (2013) identified between 11.2 and 9.0 night wakings at 6 and 12 weeks, respectively, using actigraphy. None of the diary studies included here reported > 5 wakings per night at any timepoint.

In our analysis, diary and actigraphy studies tended to be more closely aligned for TNS than for other metrics. Movement thresholds and associated algorithms employed in some actigraphy studies may be biased, consistently inflating NW counts (Kales and Rechtschaffen, 1968) and shortening LSS durations compared with sleep diaries. Algorithm adjustments that would impact NW would be expected to directly impact LSS, with TNS being more resistant to changes in the number of sleep/wake transitions, provided that the durations of any artifactual awakenings are short, and periods of extended wakefulness are not missed.

### 4.4 Conclusion

This analysis of over two decades of aggregated data provides a convenient reference for future work seeking to quantify infant TNS, LSS, or NW. Curves constructed to describe developmental trajectories of these metrics revealed common trends but also highlighted differences that exist between key measurement methodologies (actigraphy versus parent sleep diaries).

Development of infant sleep over time has not been a common target of investigation using these nightly monitoring methods. Individual studies were often small and observed only a few selected ages. Of 35 included studies, 28 reported objective data for either a single (*N* = 14) or two time points only (*N* = 14). Aggregation of these studies revealed systematic differences between methods, most notably for metrics that focus on transitions between sleep and wakefulness. Actigraphy may detect night wakings that do not gain the attention of caregivers, but can underestimate the LSS and overestimate the frequency of NW. On the other hand, diaries monitor infant state through caregiver awareness or interaction, and thus may overestimate LSS and underestimate NW.

When considering generalizability or accuracy, it is important to note that sleep metrics depend on analytical/methodological choices, and parental behaviors like feeding methods; all of which can impact within-study variability (Rudzik et al., 2018; Tikotzky et al., 2010), and are interdependent. Information from additional metrics, for example sleep onset latency, may have utility as a correlate of TNS and safe sleep practices (Massare et al., 2024), while also being less prone to accumulation of errors in sleep/wake detection over an entire night. Sleep onset latency is often estimated from broad surveys like BISQ (Mindell et al., 2019), but is less commonly monitored nightly using diary or actigraphic methods. It was present in only three of the manuscripts included in this review (Cubero et al., 2005; Figueiredo et al., 2017; Figueiredo et al., 2016). In this review, we have not included data from sleep questionnaires, as these instruments ask for estimates spanning multiple days/weeks, and may lack psychometric robustness (Antsygina et al., 2025).

### 4.5 Future directions

Scientific resolution of the standard infant sleep trajectories will be improved through the use of digital journaling and new sensor and camera technologies capable of continuously tracking specific, objective sleep metrics. As these new methods emerge, attention should be given to standardization of methodologies, as well as the practical realities of infant monitoring. For example, if true physiological sleep is of interest, monitoring breathing and heart rate may greatly improve validity. On the other hand, caregiver mental health, adherence to safe sleep guidelines, dyadic relationships, among others, may correlate more directly with interaction-associated factors like attention-seeking, sleep onset latency (at bedtime or after night waking), or frequency of caregiver intervention. Similarly, the intensity, duration, consolability, or predictability of crying may be especially informative, beyond, for example, a simple count of wakings. Monitoring multiple sleep metrics over time would be relevant for better understanding the impact of conditions that disturb sleep and settling, such as Down syndrome, autism spectrum disorder, or fetal alcohol spectrum disorder (Kamara and Beauchaine, 2020). While further research will be required to optimize infant sleep monitoring, existing technologies are poised to yield new insights correcting assumptions previously made that were hindered by issues of scale, subjectivity, memory, and/or resolution.
