# Supplementary material for: Maturation of infant sleep during the first 6 months of life: a mini-scoping review
Source: Front Neurosci. 2025 Apr 30;19:1581325. doi: 10.3389/fnins.2025.1581325 (PMC12075199; doi:10.3389/fnins.2025.1581325)
Supplement: Supplementary file 1 [file Table_1.docx]

Supplemental Table 1: Data Associated with Figure 1. Variability metrics: 1 = standard deviation, 2 = standard error, 3 = 95% CI, 4 = IQR, 5 = Range

| Author, Year | Mode | Metric | Weeks | Value | Sample Size | Corrected Sample Size | Variability | Variablity Metric |
| --- | --- | --- | --- | --- | --- | --- | --- | --- |
| Adams El , 2019 | Actigraphy | TNS | 6 | 8.6 | 23 | 24 | 1.3 | 1 |
| Adams El , 2019 | Actigraphy | TNS | 15 | 8.8 | 24 | 24 | 0.9 | 1 |
| Adams El , 2019 | Actigraphy | TNS | 24 | 9 | 23 | 24 | 0.7 | 1 |
| Ball HL, 2003 | Diary | NW | 4 | 2.41 | 253 | 253 | NR | NR |
| Ball HL, 2003 | Diary | NW | 12 | 1.63 | 248 | 248 | NR | NR |
| Camerota M, 2018 | Actigraphy | TNS | 12 | 7.56 | 82 | 82 | 1.53 | 1 |
| Camerota M, 2018 | Actigraphy | NW | 12 | 2.5 | 82 | 103 | 1.52 | 1 |
| Camerota M, 2018 | Actigraphy | LSS | 12 | 4.47 | 82 | 82 | 1.83 | 1 |
| Camerota M, 2018 | Diary | NW | 12 | 2.18 | 87 | 87 | 1.3 | 1 |
| Colombo J, 2021 | Actigraphy | TNS | 2 | 7.1 | 66 | 16.5 | 0.2 | 2 |
| Colombo J, 2021 | Actigraphy | TNS | 3 | 7.1 | 66 | 16.5 | 0.2 | 2 |
| Colombo J, 2021 | Actigraphy | TNS | 4 | 7.1 | 66 | 16.5 | 0.2 | 2 |
| Colombo J, 2021 | Actigraphy | TNS | 5 | 7.1 | 66 | 16.5 | 0.2 | 2 |
| Colombo J, 2021 | Actigraphy | TNS | 10 | 6.4 | 66 | 33 | 0.2 | 2 |
| Colombo J, 2021 | Actigraphy | TNS | 11 | 6.4 | 66 | 33 | 0.2 | 2 |
| Colombo J, 2021 | Actigraphy | TNS | 16 | 7.3 | 66 | 33 | 0.1 | 2 |
| Colombo J, 2021 | Actigraphy | TNS | 17 | 7.3 | 66 | 33 | 0.1 | 2 |
| Colombo J, 2021 | Actigraphy | NW | 2 | 3.3 | 66 | 16.5 | 0.1 | 2 |
| Colombo J, 2021 | Actigraphy | NW | 3 | 3.3 | 66 | 16.5 | 0.1 | 2 |
| Colombo J, 2021 | Actigraphy | NW | 4 | 3.3 | 66 | 16.5 | 0.1 | 2 |
| Colombo J, 2021 | Actigraphy | NW | 5 | 3.3 | 66 | 16.5 | 0.1 | 2 |
| Colombo J, 2021 | Actigraphy | NW | 10 | 3.6 | 66 | 33 | 0.2 | 2 |
| Colombo J, 2021 | Actigraphy | NW | 11 | 3.6 | 66 | 33 | 0.2 | 2 |
| Colombo J, 2021 | Actigraphy | NW | 16 | 3.7 | 66 | 33 | 0.2 | 2 |
| Colombo J, 2021 | Actigraphy | NW | 17 | 3.7 | 66 | 33 | 0.2 | 2 |
| Colombo J, 2021 | Actigraphy | LSS | 2 | 2.4 | 66 | 16.5 | 0.1 | 2 |
| Colombo J, 2021 | Actigraphy | LSS | 3 | 2.4 | 66 | 16.5 | 0.1 | 2 |
| Colombo J, 2021 | Actigraphy | LSS | 4 | 2.4 | 66 | 16.5 | 0.1 | 2 |
| Colombo J, 2021 | Actigraphy | LSS | 5 | 2.4 | 66 | 16.5 | 0.1 | 2 |
| Colombo J, 2021 | Actigraphy | LSS | 10 | 2.25 | 66 | 33 | 0.15 | 2 |
| Colombo J, 2021 | Actigraphy | LSS | 11 | 2.25 | 66 | 33 | 0.15 | 2 |
| Colombo J, 2021 | Actigraphy | LSS | 16 | 2.5 | 66 | 33 | 0.15 | 2 |
| Colombo J, 2021 | Actigraphy | LSS | 17 | 2.5 | 66 | 33 | 0.15 | 2 |
| Cubero J, 2005 | Actigraphy | TNS | 12 | 7.12 | 8 | 8 | 0.72 | 1 |
| Cubero J, 2005 | Actigraphy | TNS | 12 | 8.5 | 8 | 8 | 0.82 | 1 |
| Figueiredo B, 2016 | Diary | TNS | 2 | 6.91 | 94 | 94 | 1.4 | 1 |
| Figueiredo B, 2016 | Diary | LSS | 2 | 3.04 | 94 | 94 | 1.12 | 1 |
| Figueiredo B, 2016 | Diary | NW | 2 | 2.95 | 94 | 94 | 0.81 | 1 |
| Figueiredo B, 2016 | Diary | TNS | 12 | 8.01 | 94 | 94 | 1.48 | 1 |
| Figueiredo B, 2016 | Diary | LSS | 12 | 5.24 | 94 | 94 | 2.11 | 1 |
| Figueiredo B, 2016 | Diary | NW | 12 | 2.24 | 94 | 94 | 1.07 | 1 |
| Figueiredo B, 2016 | Diary | TNS | 24 | 8.2 | 94 | 94 | 1.78 | 1 |
| Figueiredo B, 2016 | Diary | LSS | 24 | 5.56 | 94 | 94 | 2.6 | 1 |
| Figueiredo B, 2016 | Diary | NW | 24 | 2.11 | 94 | 94 | 1.05 | 1 |
| Figueiredo B, 2017 | Diary | TNS | 2 | 6.88 | 148 | 148 | 1.38 | 1 |
| Figueiredo B, 2017 | Diary | TNS | 12 | 8.11 | 162 | 162 | 1.29 | 1 |
| Figueiredo B, 2017 | Diary | TNS | 24 | 8.4 | 148 | 123 | 1.31 | 1 |
| Figueiredo B, 2017 | Diary | NW | 2 | 2.93 | 148 | 148 | 0.92 | 1 |
| Figueiredo B, 2017 | Diary | NW | 12 | 2.08 | 162 | 162 | 1.17 | 1 |
| Figueiredo B, 2017 | Diary | NW | 24 | 2.02 | 148 | 123 | 1.01 | 1 |
| Figueiredo B, 2017 | Diary | LSS | 2 | 3.07 | 148 | 148 | 3.07 | 1 |
| Figueiredo B, 2017 | Diary | LSS | 12 | 5.6 | 162 | 162 | 2.28 | 1 |
| Figueiredo B, 2017 | Diary | LSS | 24 | 5.88 | 148 | 123 | 2.49 | 1 |
| Galland BC, 2016 | Actigraphy | TNS | 24 | 8.83 | 39 | 39 | 0.28 | 1 |
| Galland BC, 2016 | Actigraphy | NW | 24 | 2.7 | 39 | 39 | 0.3 | 1 |
| Galland BC, 2016 | Diary | TNS | 24 | 10.37 | 39 | 39 | 0.18 | 1 |
| Galland BC, 2016 | Diary | NW | 24 | 1.8 | 39 | 39 | 0.2 | 1 |
| Galland BC, 2017 | Actigraphy | TNS | 24 | 8.98 | 209 | 209 | 1.76 | 1 |
| Galland BC, 2017 | Actigraphy | NW | 24 | 2.6 | 209 | 209 | 1.6 | 1 |
| Galland BC, 2017 | Diary | TNS | 24 | 10.9 | 209 | 209 | 1.4 | 1 |
| Galland BC, 2017 | Diary | NW | 24 | 1.5 | 209 | 209 | 1.5 | 1 |
| Galland BC, 2017 | Diary | LSS | 24 | 7.4 | 209 | 209 | 2.8 | 1 |
| Guyer C, 2015 | Diary | TNS | 5 | 7.7 | 14 | 34 | 0.2 | 2 |
| Guyer C, 2015 | Diary | TNS | 11 | 8.6 | 14 | 34 | 0.1 | 2 |
| Guyer C, 2015 | Diary | TNS | 5 | 8.3 | 34 | 34 | 0.1 | 2 |
| Guyer C, 2015 | Diary | TNS | 11 | 9.2 | 34 | 34 | 0.1 | 2 |
| Guyer C, 2015 | Diary | LSS | 5 | 3.14 | 14 | 14 | 0.19 | 2 |
| Guyer C, 2015 | Diary | LSS | 11 | 4.05 | 14 | 14 | 0.17 | 2 |
| Guyer C, 2015 | Diary | LSS | 5 | 3.82 | 34 | 34 | 0.16 | 2 |
| Guyer C, 2015 | Diary | LSS | 11 | 4.73 | 34 | 34 | 0.14 | 2 |
| Hauck JL, 2018 | Actigraphy | TNS | 24 | 9.52 | 22 | 22 | 1.39 | 1 |
| Konrad C, 2016 | Actigraphy | TNS | 24 | 10.44 | 24 | 24 | 1.35 | 1 |
| Konrad C, 2016 | Actigraphy | NW | 24 | 1.9 | 24 | 24 | 1.4 | 1 |
| Pennestri MH, 2020 | Diary | NW | 24 | 2.31 | 44 | 44 | 1.16 | 1 |
| Pennestri MH, 2020 | Diary | LSS | 24 | 5.68 | 44 | 44 | 2.24 | 1 |
| Pisch M, 2019 | Actigraphy | TNS | 16 | 10.02 | 40 | 40 | 1.11 | 1 |
| Pisch M, 2019 | Actigraphy | TNS | 24 | 10.5 | 40 | 40 | 0.77 | 1 |
| Pisch M, 2019 | Actigraphy | NW | 16 | 3.74 | 40 | 40 | 1.15 | 1 |
| Pisch M, 2019 | Actigraphy | NW | 24 | 2.81 | 40 | 40 | 1 | 1 |
| Quillin SIM, 2004 | Diary | TNS | 4 | 6.4 | 13 | 13 | 1 | 1 |
| Quillin SIM, 2004 | Diary | TNS | 4 | 6.4 | 20 | 20 | 0.8 | 1 |
| Quillin SIM, 2004 | Diary | NW | 4 | 2.2 | 13 | 13 | 0.8 | 1 |
| Quillin SIM, 2004 | Diary | NW | 4 | 2 | 20 | 20 | 0.9 | 1 |
| Rudzik AEF, 2018 | Actigraphy | TNS | 4 | 8.09 | 16 | 16 | NR | NR |
| Rudzik AEF, 2018 | Actigraphy | TNS | 6 | 8.18 | 20 | 20 | NR | NR |
| Rudzik AEF, 2018 | Actigraphy | TNS | 8 | 8.69 | 19 | 19 | NR | NR |
| Rudzik AEF, 2018 | Actigraphy | TNS | 10 | 8.62 | 16 | 16 | NR | NR |
| Rudzik AEF, 2018 | Actigraphy | TNS | 12 | 9.21 | 15 | 15 | NR | NR |
| Rudzik AEF, 2018 | Actigraphy | TNS | 14 | 9.21 | 14 | 14 | NR | NR |
| Rudzik AEF, 2018 | Actigraphy | TNS | 16 | 10.1 | 13 | 13 | NR | NR |
| Rudzik AEF, 2018 | Actigraphy | TNS | 18 | 10.5 | 15 | 15 | NR | NR |
| Rudzik AEF, 2018 | Actigraphy | TNS | 4 | 8.34 | 24 | 24 | NR | NR |
| Rudzik AEF, 2018 | Actigraphy | TNS | 6 | 7.89 | 27 | 27 | NR | NR |
| Rudzik AEF, 2018 | Actigraphy | TNS | 8 | 8.25 | 26 | 26 | NR | NR |
| Rudzik AEF, 2018 | Actigraphy | TNS | 10 | 8.88 | 26 | 26 | NR | NR |
| Rudzik AEF, 2018 | Actigraphy | TNS | 12 | 9.16 | 22 | 22 | NR | NR |
| Rudzik AEF, 2018 | Actigraphy | TNS | 14 | 9.41 | 20 | 20 | NR | NR |
| Rudzik AEF, 2018 | Actigraphy | TNS | 16 | 9.59 | 20 | 20 | NR | NR |
| Rudzik AEF, 2018 | Actigraphy | TNS | 18 | 9.21 | 24 | 24 | NR | NR |
| Rudzik AEF, 2018 | Actigraphy | LSS | 4 | 2.18 | 16 | 16 | NR | NR |
| Rudzik AEF, 2018 | Actigraphy | LSS | 6 | 2.28 | 20 | 20 | NR | NR |
| Rudzik AEF, 2018 | Actigraphy | LSS | 8 | 2.43 | 19 | 19 | NR | NR |
| Rudzik AEF, 2018 | Actigraphy | LSS | 10 | 2.14 | 16 | 16 | NR | NR |
| Rudzik AEF, 2018 | Actigraphy | LSS | 12 | 2.56 | 15 | 15 | NR | NR |
| Rudzik AEF, 2018 | Actigraphy | LSS | 14 | 2.37 | 14 | 14 | NR | NR |
| Rudzik AEF, 2018 | Actigraphy | LSS | 16 | 2.46 | 13 | 13 | NR | NR |
| Rudzik AEF, 2018 | Actigraphy | LSS | 18 | 3.26 | 15 | 15 | NR | NR |
| Rudzik AEF, 2018 | Actigraphy | LSS | 4 | 2.06 | 24 | 24 | NR | NR |
| Rudzik AEF, 2018 | Actigraphy | LSS | 6 | 2.38 | 27 | 27 | NR | NR |
| Rudzik AEF, 2018 | Actigraphy | LSS | 8 | 2.28 | 26 | 26 | NR | NR |
| Rudzik AEF, 2018 | Actigraphy | LSS | 10 | 2.32 | 26 | 26 | NR | NR |
| Rudzik AEF, 2018 | Actigraphy | LSS | 12 | 2.5 | 22 | 22 | NR | NR |
| Rudzik AEF, 2018 | Actigraphy | LSS | 14 | 2.41 | 20 | 20 | NR | NR |
| Rudzik AEF, 2018 | Actigraphy | LSS | 16 | 2.85 | 20 | 20 | NR | NR |
| Rudzik AEF, 2018 | Actigraphy | LSS | 18 | 2.33 | 24 | 24 | NR | NR |
| Rudzik AEF, 2018 | Actigraphy | NW | 4 | 9.9 | 16 | 16 | NR | NR |
| Rudzik AEF, 2018 | Actigraphy | NW | 6 | 10.7 | 20 | 20 | NR | NR |
| Rudzik AEF, 2018 | Actigraphy | NW | 8 | 9.7 | 19 | 19 | NR | NR |
| Rudzik AEF, 2018 | Actigraphy | NW | 10 | 8.6 | 16 | 16 | NR | NR |
| Rudzik AEF, 2018 | Actigraphy | NW | 12 | 9 | 15 | 15 | NR | NR |
| Rudzik AEF, 2018 | Actigraphy | NW | 14 | 7.4 | 14 | 14 | NR | NR |
| Rudzik AEF, 2018 | Actigraphy | NW | 16 | 5.9 | 13 | 13 | NR | NR |
| Rudzik AEF, 2018 | Actigraphy | NW | 18 | 6.6 | 15 | 15 | NR | NR |
| Rudzik AEF, 2018 | Actigraphy | NW | 4 | 9.5 | 24 | 24 | NR | NR |
| Rudzik AEF, 2018 | Actigraphy | NW | 6 | 9.2 | 27 | 27 | NR | NR |
| Rudzik AEF, 2018 | Actigraphy | NW | 8 | 9.8 | 26 | 26 | NR | NR |
| Rudzik AEF, 2018 | Actigraphy | NW | 10 | 9.3 | 26 | 26 | NR | NR |
| Rudzik AEF, 2018 | Actigraphy | NW | 12 | 8.2 | 22 | 22 | NR | NR |
| Rudzik AEF, 2018 | Actigraphy | NW | 14 | 8.8 | 20 | 20 | NR | NR |
| Rudzik AEF, 2018 | Actigraphy | NW | 16 | 8 | 20 | 20 | NR | NR |
| Rudzik AEF, 2018 | Actigraphy | NW | 18 | 7.6 | 24 | 24 | NR | NR |
| Rudzik AEF, 2018 | Diary | TNS | 4 | 8.27 | 17 | 17 | NR | NR |
| Rudzik AEF, 2018 | Diary | TNS | 6 | 8.11 | 19 | 19 | NR | NR |
| Rudzik AEF, 2018 | Diary | TNS | 8 | 8.88 | 20 | 20 | NR | NR |
| Rudzik AEF, 2018 | Diary | TNS | 10 | 9.15 | 17 | 17 | NR | NR |
| Rudzik AEF, 2018 | Diary | TNS | 12 | 9.57 | 17 | 17 | NR | NR |
| Rudzik AEF, 2018 | Diary | TNS | 14 | 9.33 | 16 | 16 | NR | NR |
| Rudzik AEF, 2018 | Diary | TNS | 16 | 9.71 | 14 | 14 | NR | NR |
| Rudzik AEF, 2018 | Diary | TNS | 18 | 10.2 | 16 | 16 | NR | NR |
| Rudzik AEF, 2018 | Diary | TNS | 4 | 8.54 | 29 | 29 | NR | NR |
| Rudzik AEF, 2018 | Diary | TNS | 6 | 8.38 | 28 | 28 | NR | NR |
| Rudzik AEF, 2018 | Diary | TNS | 8 | 9.53 | 29 | 29 | NR | NR |
| Rudzik AEF, 2018 | Diary | TNS | 10 | 10.05 | 28 | 28 | NR | NR |
| Rudzik AEF, 2018 | Diary | TNS | 12 | 10.25 | 29 | 29 | NR | NR |
| Rudzik AEF, 2018 | Diary | TNS | 14 | 10.29 | 27 | 27 | NR | NR |
| Rudzik AEF, 2018 | Diary | TNS | 16 | 10.47 | 25 | 25 | NR | NR |
| Rudzik AEF, 2018 | Diary | TNS | 18 | 10.29 | 25 | 25 | NR | NR |
| Rudzik AEF, 2018 | Diary | LSS | 4 | 3.12 | 17 | 17 | NR | NR |
| Rudzik AEF, 2018 | Diary | LSS | 6 | 3.58 | 19 | 19 | NR | NR |
| Rudzik AEF, 2018 | Diary | LSS | 8 | 4.53 | 20 | 20 | NR | NR |
| Rudzik AEF, 2018 | Diary | LSS | 10 | 5.07 | 17 | 17 | NR | NR |
| Rudzik AEF, 2018 | Diary | LSS | 12 | 5.31 | 17 | 17 | NR | NR |
| Rudzik AEF, 2018 | Diary | LSS | 14 | 5.42 | 16 | 16 | NR | NR |
| Rudzik AEF, 2018 | Diary | LSS | 16 | 5.3 | 14 | 14 | NR | NR |
| Rudzik AEF, 2018 | Diary | LSS | 18 | 6.28 | 16 | 16 | NR | NR |
| Rudzik AEF, 2018 | Diary | LSS | 4 | 3.33 | 29 | 29 | NR | NR |
| Rudzik AEF, 2018 | Diary | LSS | 6 | 4.1 | 28 | 28 | NR | NR |
| Rudzik AEF, 2018 | Diary | LSS | 8 | 4.98 | 29 | 29 | NR | NR |
| Rudzik AEF, 2018 | Diary | LSS | 10 | 6.71 | 28 | 28 | NR | NR |
| Rudzik AEF, 2018 | Diary | LSS | 12 | 7.39 | 29 | 29 | NR | NR |
| Rudzik AEF, 2018 | Diary | LSS | 14 | 7.46 | 27 | 27 | NR | NR |
| Rudzik AEF, 2018 | Diary | LSS | 16 | 7.59 | 25 | 25 | NR | NR |
| Rudzik AEF, 2018 | Diary | LSS | 18 | 7.88 | 25 | 25 | NR | NR |
| Rudzik AEF, 2018 | Diary | NW | 4 | 4.6 | 17 | 17 | NR | NR |
| Rudzik AEF, 2018 | Diary | NW | 6 | 4.5 | 19 | 19 | NR | NR |
| Rudzik AEF, 2018 | Diary | NW | 8 | 3.7 | 20 | 20 | NR | NR |
| Rudzik AEF, 2018 | Diary | NW | 10 | 3.3 | 17 | 17 | NR | NR |
| Rudzik AEF, 2018 | Diary | NW | 12 | 2.8 | 17 | 17 | NR | NR |
| Rudzik AEF, 2018 | Diary | NW | 14 | 3.2 | 16 | 16 | NR | NR |
| Rudzik AEF, 2018 | Diary | NW | 16 | 2.6 | 14 | 14 | NR | NR |
| Rudzik AEF, 2018 | Diary | NW | 18 | 1.9 | 16 | 16 | NR | NR |
| Rudzik AEF, 2018 | Diary | NW | 4 | 3.9 | 29 | 29 | NR | NR |
| Rudzik AEF, 2018 | Diary | NW | 6 | 3.1 | 28 | 28 | NR | NR |
| Rudzik AEF, 2018 | Diary | NW | 8 | 3.1 | 29 | 29 | NR | NR |
| Rudzik AEF, 2018 | Diary | NW | 10 | 2.3 | 28 | 28 | NR | NR |
| Rudzik AEF, 2018 | Diary | NW | 12 | 2 | 29 | 29 | NR | NR |
| Rudzik AEF, 2018 | Diary | NW | 14 | 1.9 | 27 | 27 | NR | NR |
| Rudzik AEF, 2018 | Diary | NW | 16 | 1.8 | 25 | 25 | NR | NR |
| Rudzik AEF, 2018 | Diary | NW | 18 | 1.7 | 25 | 25 | NR | NR |
| Santos IS, 2019 | Actigraphy | TNS | 12 | 8.03 | 230 | 230 | 1.48 | 1 |
| Santos IS, 2019 | Actigraphy | TNS | 24 | 8.59 | 214 | 214 | 1.33 | 1 |
| Santos IS, 2019 | Actigraphy | NW | 12 | 3.3 | 230 | 230 | 2.5,4.0 | 4 |
| Santos IS, 2019 | Actigraphy | NW | 24 | 2 | 214 | 214 | 1.7,3.0 | 4 |
| Santos IS, 2019 | Diary | TNS | 12 | 9.53 | 258 | 258 | 2.07 | 1 |
| Santos IS, 2019 | Diary | TNS | 24 | 9.49 | 240 | 240 | 2.07 | 1 |
| Santos IS, 2019 | Diary | NW | 12 | 2 | 258 | 258 | 1.3,3.0 | 4 |
| Santos IS, 2019 | Diary | NW | 24 | 1.7 | 240 | 240 | 1.0,2.7 | 4 |
| Scher A, 2005 | Actigraphy | TNS | 12 | 8.87 | 50 | 50 | 1.31 | 1 |
| Scher A, 2005 | Actigraphy | LSS | 12 | 1.85 | 50 | 50 | 0.94 | 1 |
| Scher A, 2005 | Actigraphy | NW | 12 | 1.95 | 50 | 50 | 1.56 | 1 |
| Scher A, 2005 | Actigraphy | TNS | 24 | 8.6 | 37 | 37 | 1.18 | 1 |
| Scher A, 2005 | Actigraphy | LSS | 24 | 1.21 | 37 | 37 | 0.46 | 1 |
| Scher A, 2005 | Actigraphy | NW | 24 | 3.3 | 37 | 37 | 1.85 | 1 |
| Scher A, 2015 | Actigraphy | TNS | 20 | 9.83 | 26 | 26 | 0.85 | 1 |
| Scher A, 2015 | Actigraphy | NW | 20 | 2.3 | 26 | 26 | 0.92 | 1 |
| Scher A, 2015 | Actigraphy | TNS | 24 | 10.32 | 26 | 26 | 0.97 | 1 |
| Scher A, 2015 | Actigraphy | NW | 24 | 2.37 | 26 | 26 | 0.96 | 1 |
| Shinohara H, 2012 | Actigraphy | TNS | 4 | 8.2 | 26 | 8.67 | 1.04 | 1 |
| Shinohara H, 2012 | Actigraphy | TNS | 5 | 8.2 | 26 | 8.67 | 1.04 | 1 |
| Shinohara H, 2012 | Actigraphy | TNS | 6 | 8.2 | 26 | 8.67 | 1.04 | 1 |
| Shinohara H, 2012 | Actigraphy | TNS | 8 | 8.58 | 26 | 8.67 | 1.5 | 1 |
| Shinohara H, 2012 | Actigraphy | TNS | 9 | 8.58 | 26 | 8.67 | 1.5 | 1 |
| Shinohara H, 2012 | Actigraphy | TNS | 10 | 8.58 | 26 | 8.67 | 1.5 | 1 |
| Shinohara H, 2012 | Actigraphy | TNS | 14 | 9.35 | 26 | 8.67 | 1.48 | 1 |
| Shinohara H, 2012 | Actigraphy | TNS | 15 | 9.35 | 26 | 8.67 | 1.48 | 1 |
| Shinohara H, 2012 | Actigraphy | TNS | 16 | 9.35 | 26 | 8.67 | 1.48 | 1 |
| Spruyt K, 2008 | Actigraphy | TNS | 12 | 8.09 | 17 | 17 | 1.54 | 1 |
| Spruyt K, 2008 | Actigraphy | TNS | 24 | 9.46 | 18 | 18 | 1.13 | 1 |
| Spruyt K, 2008 | Diary | TNS | 12 | 9.58 | 17 | 17 | 0.94 | 1 |
| Spruyt K, 2008 | Diary | TNS | 24 | 9.66 | 18 | 18 | 1.12 | 1 |
| St James-Roberts I, 2001 | Diary | TNS | 3 | 8.2 | 203 | 203 | 8.03, 8.37 | 3 |
| St James-Roberts I, 2001 | Diary | TNS | 6 | 8.43 | 203 | 203 | 8.25, 8.61 | 3 |
| St James-Roberts I, 2001 | Diary | TNS | 12 | 9.33 | 203 | 203 | 9.15,9.51 | 3 |
| St James-Roberts I, 2001 | Diary | NW | 3 | 3.2 | 203 | 203 | 3.0,3.4 | 3 |
| St James-Roberts I, 2001 | Diary | NW | 6 | 2.6 | 203 | 203 | 2.4,2.8 | 3 |
| St James-Roberts I, 2001 | Diary | NW | 12 | 1.8 | 203 | 203 | 1.6, 2.0 | 3 |
| Stremler R, 2006 | Actigraphy | TNS | 6 | 7.46 | 15 | 15 | 6.97, 7.97 | 3 |
| Stremler R, 2006 | Actigraphy | NW | 6 | 12.3 | 15 | 15 | 10.1, 14.6 | 3 |
| Stremler R, 2006 | Actigraphy | LSS | 6 | 2.85 | 15 | 15 | 2.3, 3.4 | 3 |
| Stremler R, 2013 | Actigraphy | TNS | 6 | 7.63 | 103 | 103 | 0.85 | 1 |
| Stremler R, 2013 | Actigraphy | TNS | 12 | 8.68 | 102 | 102 | 0.93 | 1 |
| Stremler R, 2013 | Actigraphy | NW | 6 | 11.2 | 103 | 103 | 3.4 | 1 |
| Stremler R, 2013 | Actigraphy | NW | 12 | 9 | 102 | 102 | 4.1 | 1 |
| Stremler R, 2013 | Actigraphy | LSS | 6 | 2.82 | 103 | 103 | 0.75 | 1 |
| Stremler R, 2013 | Actigraphy | LSS | 12 | 4 | 102 | 102 | 1.45 | 1 |
| Sweeney BM, 2020 | Actigraphy | TNS | 6 | 9.16 | 20 | 20 | 0.79 | 1 |
| Sweeney BM, 2020 | Actigraphy | TNS | 12 | 9.78 | 20 | 20 | 0.8 | 1 |
| Sweeney BM, 2020 | Actigraphy | LSS | 6 | 4.47 | 20 | 20 | 0.95 | 1 |
| Sweeney BM, 2020 | Actigraphy | LSS | 12 | 5.91 | 20 | 20 | 1.63 | 1 |
| Sweeney BM, 2020 | Actigraphy | NW | 6 | 3.5 | 20 | 20 | 3, 5 | 5 |
| Sweeney BM, 2020 | Actigraphy | NW | 12 | 3 | 20 | 20 | 2, 4.5 | 5 |
| Symon BG, 2005 | Actigraphy | TNS | 6 | 8.1 | 121 | 121 | NR | NR |
| Symon BG, 2005 | Actigraphy | LSS | 6 | 4.8 | 121 | 121 | NR | NR |
| Symon BG, 2005 | Actigraphy | TNS | 12 | 8.7 | 107 | 107 | NR | NR |
| Symon BG, 2005 | Actigraphy | LSS | 12 | 6.3 | 107 | 107 | NR | NR |
| Tikotzky L, 2009 | Actigraphy | TNS | 24 | 9.82 | 85 | 85 | 1.03 | 1 |
| Tikotzky L, 2009 | Actigraphy | NW | 24 | 1.69 | 85 | 85 | 1.05 | 1 |
| Tikotzky L, 2009 | Diary | NW | 24 | 2.67 | 85 | 85 | 1.91 | 1 |
| Tikotzky L, 2010 | Actigraphy | TNS | 24 | 9.8 | 96 | 96 | 0.95 | 1 |
| Tikotzky L, 2010 | Actigraphy | NW | 24 | 1.79 | 96 | 96 | 1.06 | 1 |
| Tikotzky L, 2015 | Actigraphy | TNS | 12 | 9.45 | 56 | 56 | 1.14 | 1 |
| Tikotzky L, 2015 | Actigraphy | TNS | 24 | 9.67 | 54 | 54 | 0.83 | 1 |
| Tikotzky L, 2015 | Actigraphy | NW | 12 | 2.49 | 56 | 56 | 0.98 | 1 |
| Tikotzky L, 2015 | Actigraphy | NW | 24 | 2.19 | 54 | 54 | 1.14 | 1 |
| Tikotzky L, 2015 | Diary | NW | 12 | 2.52 | 56 | 56 | 1.17 | 1 |
| Tikotzky L, 2015 | Diary | NW | 24 | 3.17 | 54 | 54 | 1.74 | 1 |
| Tsai Sy, 2018 | Actigraphy | TNS | 24 | 7.8 | 118 | 118 | 0.89 | 1 |
| Tsai Sy, 2018 | Actigraphy | TNS | 24 | 7.71 | 69 | 69 | 1.09 | 1 |
| Tsai Sy, 2018 | Actigraphy | TNS | 24 | 7.94 | 32 | 32 | 0.88 | 1 |
| Tsai Sy, 2018 | Diary | TNS | 24 | 9.01 | 118 | 118 | 1.23 | 1 |
| Tsai Sy, 2018 | Diary | TNS | 24 | 9.31 | 69 | 69 | 1.35 | 1 |
| Tsai Sy, 2018 | Diary | TNS | 24 | 8.76 | 32 | 32 | 1.24 | 1 |
| Tsai Sy, 2018 | Diary | LSS | 24 | 6.41 | 118 | 118 | 2.5 | 1 |
| Tsai Sy, 2018 | Diary | LSS | 24 | 8.42 | 69 | 69 | 2.49 | 1 |
| Tsai Sy, 2018 | Diary | LSS | 24 | 7.03 | 32 | 32 | 2.68 | 1 |
| Tsai Sy, 2018 | Diary | NW | 24 | 2 | 118 | 118 | 1.24 | 1 |
| Tsai Sy, 2018 | Diary | NW | 24 | 1.04 | 69 | 69 | 1.03 | 1 |
| Tsai Sy, 2018 | Diary | NW | 24 | 1.56 | 32 | 32 | 0.84 | 1 |
| Tsai Sy, 2022 | Actigraphy | TNS | 24 | 7.85 | 320 | 320 | 0.94 | 1 |
| Vijakkhana N, 2015 | Diary | TNS | 24 | 9.6 | 208 | 208 | 1.19 | 1 |
| Vijakkhana N, 2015 | Diary | TNS | 24 | 9.81 | 208 | 208 | 1.15 | 1 |
| Volkovich E, 2015 | Actigraphy | TNS | 12 | 9.4 | 127 | 127 | 1.21 | 1 |
| Volkovich E, 2015 | Actigraphy | TNS | 24 | 9.59 | 114 | 114 | 0.93 | 1 |
| Volkovich E, 2015 | Actigraphy | NW | 12 | 2.8 | 127 | 127 | 1.29 | 1 |
| Volkovich E, 2015 | Actigraphy | NW | 24 | 2.22 | 114 | 114 | 1.32 | 1 |
| Volkovich E, 2015 | Actigraphy | LSS | 12 | 4.26 | 127 | 127 | 1.4 | 1 |
| Volkovich E, 2015 | Actigraphy | LSS | 24 | 3.93 | 114 | 114 | 1.44 | 1 |
| Volkovich E, 2015 | Diary | NW | 12 | 2.16 | 127 | 127 | 1.24 | 1 |
| Volkovich E, 2015 | Diary | NW | 24 | 2.64 | 114 | 114 | 1.62 | 1 |
| Volkovich E, 2018 | Actigraphy | TNS | 12 | 9.29 | 127 | 127 | 1.4 | 1 |
| Volkovich E, 2018 | Actigraphy | TNS | 24 | 9.66 | 121 | 121 | 1.26 | 1 |
| Volkovich E, 2018 | Actigraphy | LSS | 12 | 4.22 | 127 | 127 | 1.4 | 1 |
| Volkovich E, 2018 | Actigraphy | LSS | 24 | 3.91 | 121 | 121 | 1.36 | 1 |
| Volkovich E, 2018 | Actigraphy | NW | 12 | 2.82 | 127 | 127 | 1.2 | 1 |
| Volkovich E, 2018 | Actigraphy | NW | 24 | 2.25 | 121 | 121 | 1.3 | 1 |
| Volkovich E, 2018 | Diary | NW | 12 | 2.24 | 127 | 127 | 1.17 | 1 |
| Volkovich E, 2018 | Diary | NW | 24 | 2.67 | 121 | 121 | 1.5 | 1 |
| Yu X, 2021 | Actigraphy | TNS | 4 | 7.68 | 306 | 306 | 7.55, 7.825 | 3 |
| Yu X, 2021 | Actigraphy | TNS | 24 | 8.78 | 306 | 306 | 8.66, 8.91 | 3 |
| Yu X, 2021 | Actigraphy | NW | 4 | 3.3 | 306 | 306 | 3.1, 3.5 | 3 |
| Yu X, 2021 | Actigraphy | NW | 24 | 1.1 | 306 | 306 | 1.0, 1.2 | 3 |
| Yu X, 2021 | Actigraphy | LSS | 4 | 4.7 | 306 | 306 | 4.52, 4.91 | 3 |
| Yu X, 2021 | Actigraphy | LSS | 24 | 7.67 | 306 | 306 | 7.49, 7.86 | 3 |
