# Supplementary material for: Maturation of infant sleep during the first 6 months of life: a mini-scoping review
Source: Front Neurosci. 2025 Apr 30;19:1581325. doi: 10.3389/fnins.2025.1581325 (PMC12075199; doi:10.3389/fnins.2025.1581325)
Supplement: Supplementary file 2 [file Table_2.docx]

Supplemental Table 2. Mini-scoping review included studies with brief description.

| Authors | Brief Description |
| --- | --- |
| Adams EL et al. 2019 | Observational study of developmental and sleep patterns across first six-months of life and compare to mother-reported perceptions of infant sleep. |
| Ball HL et al 2003 | Used sleep logs and semi-structured interviews to explore how parents responded to infant sleep patterns, management of night-time feedings, and bed-sharing. |
| Camerota M et al. 2018 | Compared sleep variables obtained from videosomnography, actigraphy, and sleep diaries for 90 Black 3-month olds. |
| Colombo J et al. 2021 | Double-blind randomized study to evaluate nutritive effects of prebiotics on infant wake behavior, physiology, and metabolic status. |
| Cubero J et al. 2005 | Used actigraphy to compare circadian rhythm of 6-sulfatoxymelatonin in bottle-fed and breast-fed infants. |
| Figueiredo B et al. 2016 | Described infant sleep-wake behaviors using 24-hour diaries and explored developmental changes from two to twenty-four weeks of age. |
| Figueiredo B et al. 2017 | Assessed sleep-wake behavior of infants using sleep-diaries as a function of feeding method (exclusive breastfeeding, partial breastfeeding, and exclusive formula fed). |
| Galland BC et al. 2016 | Cross-sectional study to determine if nap identification could be extracted from actigraphy across infant and toddler groups. |
| Galland BC et al. 2017 | Randomized controlled trial to evaluate sleep education delivered antenatally and at 3 weeks to prevent infant sleep problems at 24 weeks of age. |
| Guyer C et al. 2015 | Compared 24-h sleep-wake rhythm in preterm infants vs. term infants at corrected age. |
| Hauck JL et al. 2018 | To examine low-intensity physical activity, sleep behavior, and growth in 24-week olds. |
| Konrad C et al. 2016 | Examined sleep quality during the night and daytime naps with actigraphy and whether them preceding a learning event are related to memory encoding. |
| Pennestri MH et al. 2020 | Described night-to-night variability in consolidated sleep in 24-week old infants using sleep diaries. |
| Pisch M et al. 2019 | Examined sleep variables and their relation to developmental cognitive trajectories. |
| Quillin SIM et al. 2004 | Examined whether there is an interaction between feeding type and sleeping arrangements with postpartum sleep. |
| Rudzik AEF et al. 2018 | Examine whether night-time sleep parameters of exclusively breastfed versus exclusively formula fed infants differ using both actigraphy and sleep diaries. |
| Santos IS et al. 2019 | Assess the efficacy of an educational intervention to promote nighttime sleep duration in a sample of 12-week old infants in Brazil. |
| Scher A et al. 2004 | Examined the developmental course of sleep consolidation from infancy to preschool. |
| Scher A et al. 2015 | Examined association between onset of crawling and changes in infant sleep. |
| Shinohara H et al. 2012 | Examine the relationship between sleep development and crying episodes in a prospective longitudinal study of infants during the first 4 months of life. |
| Spruyt K et al. 2008 | Assess the relationship between development of sleep-wake patterns, temperament and overall mental, motor, and behavioral development. |
| St. James-Roberts I et al. 2001 | Studied effectiveness of behavioral program in preventing infant crying and sleeping problems during first 12 weeks of age. |
| Stremler R et al. 2006 | Pilot study to study feasibility, acceptability, and effects of maternal-infant sleep intervention on sleep outcomes in early postpartum period. |
| Stremler R et al. 2013 | Randomized controlled trial to evaluate the effectiveness of a behavioral-educational sleep intervention in improving both maternal and infant sleep. |
| Sweeney BM et al. 2020 | Pilot of controlled trial to test the acceptability and efficacy of perinatally delivered behavioral-educational sleep intervention for first-time mothers. |
| Symon BG et al. 2005 | To evaluate the effect of behavior modification program in improving sleep performance. |
| Tikotzky L et al. 2009 | Longitudinal study to assess the development of sleep patterns among infants and the relationship with maternal cognitions regarding infant sleep. |
| Tikotzky L et al. 2010 | Use objective and subjective sleep measures to explore relationship between infant sleep patterns and infant physical growth. |
| Tikotzky L et al. 2015 | Longitudinal study to examine infant and maternal sleep from 12 to 24 weeks as well as link between paternal involvement and maternal and infant sleep. |
| Tsai SY et al. 2018 | Examined sleep characteristics of infants with parent-reported sleep problems, no sleep problems, and uncertain sleep conditions. |
| Tsai SY et al. 2022 | Examine association of timing and consistency of parent bedtime routines with infant sleep duration and variability at night. |
| Vijakkhana N et al. 2015 | Investigated whether media exposure and bedroom media use are associated with night-time sleep duration. |
| Volkovich E et al. 2015 | Assess differences in objective and subjective sleep patterns between co-sleeping and solitary sleeping mother-infant dyads. |
| Volkovich E et al. 2018 | Longitudinal examination of differences in objective and subjective sleep patterns parental functioning between room-sharing and solitary sleeping mother-infant dyads. |
| Yu X et al. 2021 | Objective assessment of sleep-wake patterns in infants and differences based on racial/ethnic backgrounds and household socioeconomic status. |
