# Supplementary material for: Maturation of infant sleep during the first 6 months of life: a mini-scoping review
Source: Front Neurosci. 2025 Apr 30;19:1581325. doi: 10.3389/fnins.2025.1581325 (PMC12075199; doi:10.3389/fnins.2025.1581325)

Supplemental Figure 1: Flow diagram for scoping review process. Initial PubMed search resulted in 1,703 results and after applying filters, inclusion/exclusion criteria, and additional bibliography searches, the total eligible sample was 35 studies.

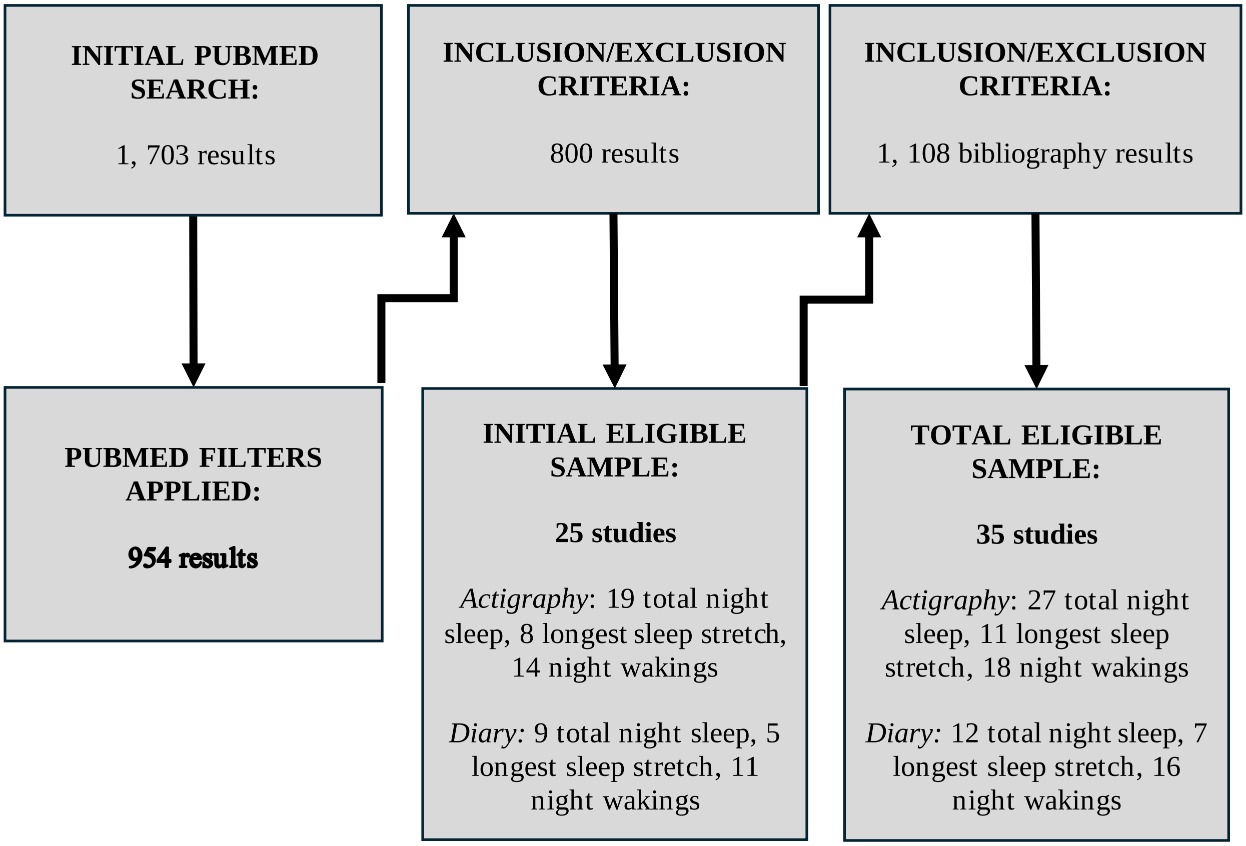

Supplement: Supplementary file 3 [file Data_Sheet_1.pdf]
